# Cannabinoid use generalizes stress responses by altering the astrocyte plasticity through extracellular matrix signaling in the nucleus accumbens core

**DOI:** 10.21203/rs.3.rs-7254957/v1

**Published:** 2025-08-21

**Authors:** Ritchy Hodebourg, Lillian Duncan, Eric Dereschewitz, Peter Kalivas

**Affiliations:** Medical University of South Carolina; Medical University of South Carolina; Medical University of South Carolina; Medical University of South Carolina

## Abstract

The rising legal acceptance of cannabis and the high comorbidity between cannabis use disorder (CUD) and post-traumatic stress disorder (PTSD) highlight the importance of understanding how stress and cannabis influence the brain. We recently discovered that cannabis use promotes two PTSD-like symptoms: avoidance coping behaviors and the generalization of stress-coping responses to a neutral stimulus not previously linked to stress. To investigate the neuroadaptations behind these changes, we used in vivo zymography and confocal microscopy to examine how stress and cannabinoid use influence multipartite synaptic plasticity in the nucleus accumbens core (NAcore), including astroglial plasticity, Synapsin-I density, and matrix metalloproteinases (MMP-2,9) activity. For this purpose, rats were restraint stressed for 2h and simultaneously exposed to an odor that became the stress-conditioned stimulus (stress-CS). Three weeks later, rats self-administered cannabinoids (delta9-tetrahydrocannabinol + cannabidiol; THC + CBD) for 10 days, followed by 10 days of withdrawal. We then evaluated the effect of stress-CS or neutral odor (NS) on coping strategies in a defensive burying task. We demonstrated for the first time that THC + CBD generalized stress responses to the NS by causing astrocytes to retract from synapses and decreasing Synapsin-I density in the NAcore. Furthermore, cannabinoid use promoted avoidant coping behaviors in response to a stress-CS by triggering strong activation of MMP-2,9, driven largely by MMP-2, causing a re-association of astrocytes to synapses along with an increase of Synapsin-I density and astrocyte atrophy. However, these neuroadaptations only occurred in males. Overall, these findings highlight potential therapeutic targets like MMPs and astrocytes for treating co-occurring CUD/PTSD.

## Introduction

For certain individuals, a single traumatic event can lead to trauma and stress-related disorders, including post-traumatic stress disorder (PTSD)[1]. Cannabis use disorder (CUD) is often comorbid with PTSD and is the most prevalent co-occurring condition among veterans diagnosed with CUD[2]. Patients with comorbid PTSD and CUD report higher PTSD symptom severity and poorer treatment outcomes relative to those without CUD[3, 4]. Furthermore, recent longitudinal studies demonstrate that cannabis use is associated with higher PTSD symptom severity, including greater traumatic thought intrusions[5, 6] and depressive symptoms[7]. Despite this evidence, PTSD is often listed as a qualified medical condition for the use of cannabis in the United States[8, 9]. As of 2025, 31 states listed cannabis as a treatment for PTSD[10]. Consequently, the increasing use of cannabis for self-medication of PTSD and the comorbid PTSD and CUD underscores the need to identify the neurobiological mechanisms through which cannabis use alters and potentially exacerbates the symptoms associated with PTSD.

Using an stress cue-conditioned (stress-CS) animal model of PTSD, we recently demonstrated that an acute stress potentiates cannabis consumption in male rats[11]. Additionally, we discovered that cannabis use promotes two significant PTSD-like symptoms: the generalization of stress-coping responses to a neutral stimulus that is not produced by stress exposure in the absence of cannabis use, and engaging in avoidance coping behaviors when faced with a conditioned stress odor[11]. Importantly, these findings differ from those observed after cocaine and heroin self-administration, where rats exposed to a stress-CS engage in active coping behaviors, without generalizing to an odor not associated with acute stress experience[12–14]. Finally, the cannabis-induced behavioral changes were accompanied by reduced spine density in the nucleus accumbens core (NAcore) and a further decrease in spine head diameter after exposure to the stress-CS[11], indicating that cannabis may be altering how environmental stimuli elicit synaptic plasticity.

Nonneuronal components of the neuropil adjacent to synapses, including astroglia and extracellular matrix (ECM), form what is termed the multipartite synapse and function together with pre- and postsynapses to regulate synaptic plasticity[15, 16]. For instance, astrocytes regulate the synaptic plasticity by insulating synapses, thereby providing them with metabolic support, gliotransmitters, and maintaining ion homeostasis[16]. In parallel, destabilizing the ECM via catalytic signaling induced by matrix metalloproteinases (MMPs) is associated with neuronal and astroglial plasticity. Both environmental stressors and addictive drugs alter ECM signaling and astroglial morphology and signaling, and contribute to the development of stress- and drug-related disorders[17]. However, the effects of cannabis use, either independently or in conjunction with stress, on the nonneuronal components of multipartite synapses remain unknown. The objective of this study was to assess whether the interaction between stress and cannabis use influences components of multipartite synapses, including astroglial plasticity, synapse density, and ECM catalytic signaling in the NAcore. For this purpose, following an acute restraint stress, astrocytes were labeled in rats that were trained to self-administer delta9-tetrahydrocannabinol + cannabidiol (THC + CBD)[18]. After assessing how cannabinoid use affected coping strategies through a defensive burying task (DBT), we employed confocal microscopy, in vivo zymography, and digital rendering techniques to quantify astrocyte morphology, the colocalization of astrocytes with the presynaptic marker Synapsin-I, and the gelatinolytic activity of MMP-2 and MMP-9 in the NAcore.

## Methods

### Animals

Male and female Long-Evans rats (~ 250 g; Charles River Laboratories) were double-housed according to sex on a 12-h light/dark cycle with food and water available ad libitum. Experiments occurred in the light cycle. All procedures received approval from the Animal Care and Use Committee of the Medical University of South Carolina and were conducted following National Institutes of Health guidelines.

### Acute stress and odor pairing

Rats were placed in a restraining device for 2 hours and paired with an odor (lemon or sandalwood; Sun essential, Phoenix, AZ, USA) that became the stress-CS. Control rats were exposed to the same odor in the home cage for 2 hours (Sham-CS). The odor that was not stress- or sham-paired was used during the DBT as a neutral odor control (stress-NS). Two weeks after the stress procedure, rats received indwelling catheters as previously described[18].

### Viral labeling

After catheter implantation, rats were implanted with bilateral guide cannulae into the NAcore (AP: 1.5mm, ML: ± 1.7mm, DV: −5.5mm). AAV5/GFAP-hM3dq-mCherry (University of Zurich) was injected into the left hemisphere (1.0 μl, 0.15 μl/min, 5 min diffusion) to label astrocytes. The microinjectors are 2mm longer than the guide cannulae to reach the target.

### THC + CBD self-administration

Following surgery, rats underwent 5 days of vapor exposure to a THC + CBD mixture in a 10:1 ratio, as previously described[18]. Rats were then trained to self-administer THC + CBD on a fixed ratio 1 schedule, during 90 min sessions for 10 consecutive days. Active lever presses delivered 0.4 + 0.04 mg/kg THC + CBD and were paired with cues (light + tone) followed by a 20-s timeout. Another cohort of rats was trained to self-administer the vehicle (1% ethanol). Following self-administration, animals underwent 10 days of withdrawal to ensure the absence of THC metabolites during the DBT.

### Defensive burying task

Coping strategies were quantified as previously described[11]. Briefly, bedding was placed in one half of the standard home cage, opposite the odor. Rats were placed facing away from the odor on the bedding side of the cage, and behavior was recorded for 15 min using Ethovision XT software (Leesburg, VA, USA). Active (burying) and avoidant (escape, immobility and grooming) coping mechanisms were quantified.

### In vivo zymography

In vivo zymography was used to quantify MMP-2,9 activity as previously described in detail[19]. FITC-gelatin was microinjected into the right hemisphere (1.5μl, 0.5μl/min, 3 min diffusion) immediately before the DBT. Rats were then perfused with 4% PFA immediately after the DBT and brains were sliced at 100μm. Only slices containing the injection track and the anterior commissure on the same frame were analyzed (ImageJ; National Institute of Mental Health, Bethesda, MD). MMP-2 (444288, MilliporeSigma) and MMP-9 (444278, MilliporeSigma) inhibitors were dissolved in 1% DMSO and bilaterally microinjected before FITC-gelatin in a separate rat cohort as previously described[20].

### Immunohistochemistry

Left hemisphere slices, containing the GFAP-mCherry virus, were permeabilized for 15 min in PBS with 0.2% Triton X-100 (PBST), then blocked with 2% PBST containing 5% normal goat serum. Rabbit anti-synapsin-I antibody (1:1000, ab64581, Abcam) was incubated for 72 hours at 4°C. Slices were washed with 0.2% PBST, then incubated with Alexa Fluor 405(1:1000, ab175652, Abcam) for 72 hours at 4°C.

### Confocal microscopy

Astrocyte images were acquired using a Leica SP5 with a 63X oil-immersion objective lens at 16-bit resolution with a 1-μm step size. Images were then imported into Imaris software (version 9.7.0). The mCherry signal was used for 3-dimensional reconstruction of astrocytes. The astrocyte-Synapsin-I proximity was quantified by using each rendered astrocyte as a region of interest (ROI) for colocalization analysis with Synapsin-I. The colocalization channel displays the results as the percentage of the ROI overlapping with Synapsin-I puncta. To determine the Synapsin-I density, we normalized the thresholded signal intensity to the volume of the frame from which it was acquired. Imaging and analysis were performed in a blinded manner.

### Statistics

Data were analyzed with GraphPad Prism 10. Behavioral and in vivo zymography data were analyzed using two-way ANOVAs with Tukey’s post hoc comparison. For astroglial measures, a D’Agostino & Pearson test was initially used to assess the normality of each group. The Kruskal-Wallis test with Dunn’s post hoc was used for non-normally distributed treatment groups. Coping strategies and astroglial distribution were analyzed with a multiple Chi-square followed by a Bonferroni correction.

## Results

### Acute stress potentiated THC + CBD use in male rats

**Figure 1:**
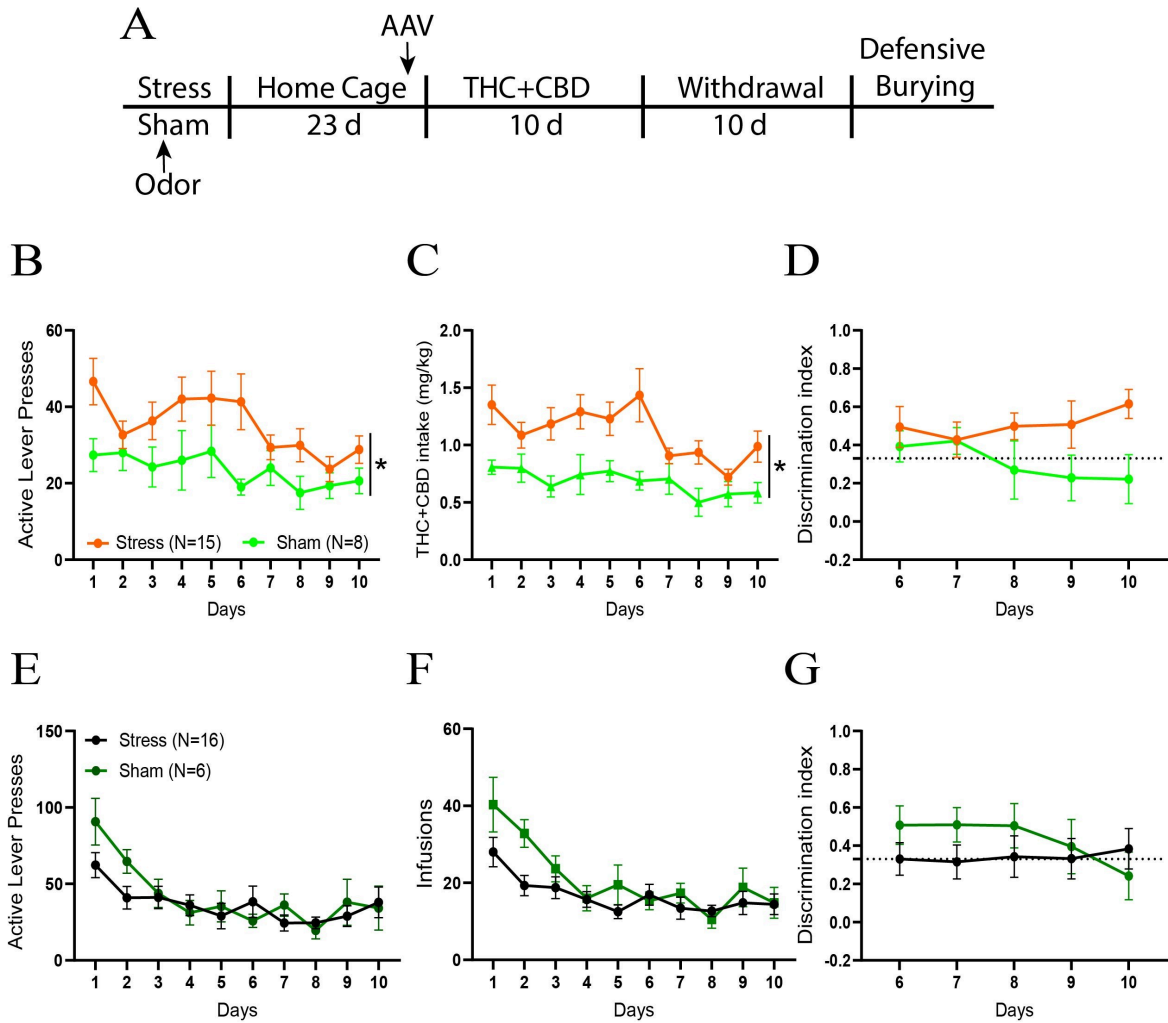
Acute restraint stress potentiated THC+CBD use in males. **A)** Experimental timeline outlining THC+CBD self-administration, withdrawal and the defensive burying task. **B)** Acute restraint stress heightened active lever pressing during THC+CBD self-administration (stress: F_1,21_=4.54, p=0.045; days: F_9,189_, p=0.002). **C)** Stressed rats consumed more THC+CBD compared to sham animals (stress: F_1,21_=5.404, p=0.03). **D)** The sham and stress groups showed no differences in their ability to distinguish between the active (THC+CBD infusion) and inactive (no consequence) levers during the final 5 days of self-administration. The dotted line represents a 2:1 ratio of active to inactive. **E)** Acute stress did not affect the active lever presses during vehicle self-administration. **F)** Sham and stress groups consumed the same amount of vehicle. **G)** No difference was observed between sham and stress rats on the discrimination index during vehicle self-administration. See table S1 for complete analysis. Data are shown as mean ± SEM. Number of animals is expressed in brackets. *p< 0.05, comparing stress to sham.

Rats were subjected to acute restraint stress for 2 hours and simultaneously exposed to an odor. 3 weeks after the stress, rats were trained to self-administer THC + CBD or its vehicle for 10 days, followed by 10 days of withdrawal (Fig. 1A). In THC + CBD trained rats, stressed males pressed more on the active lever than sham rats (Fig. 1B). Stressed males consumed more THC + CBD than sham rats (Fig. 1C). Both stressed and sham rats showed equivalent discrimination between the active and inactive levers (Fig. 1D). No differences between stress and sham groups were observed in vehicle-trained rats (Fig. 1E–G).

In female THC + CBD trained rats, no differences were measured between groups on the active lever presses and THC + CBD intake (Fig. S1A-B). However, stressed rats had a higher discrimination index than sham rats (Fig. S1C), indicating that the acute stress may increase the reinforcing value of the THC + CBD in females. No differences between stress and sham groups were observed in vehicle-trained rats (Fig. S1D-F).

### THC + CBD promoted avoidant coping strategies in male rats

**Figure 2:**
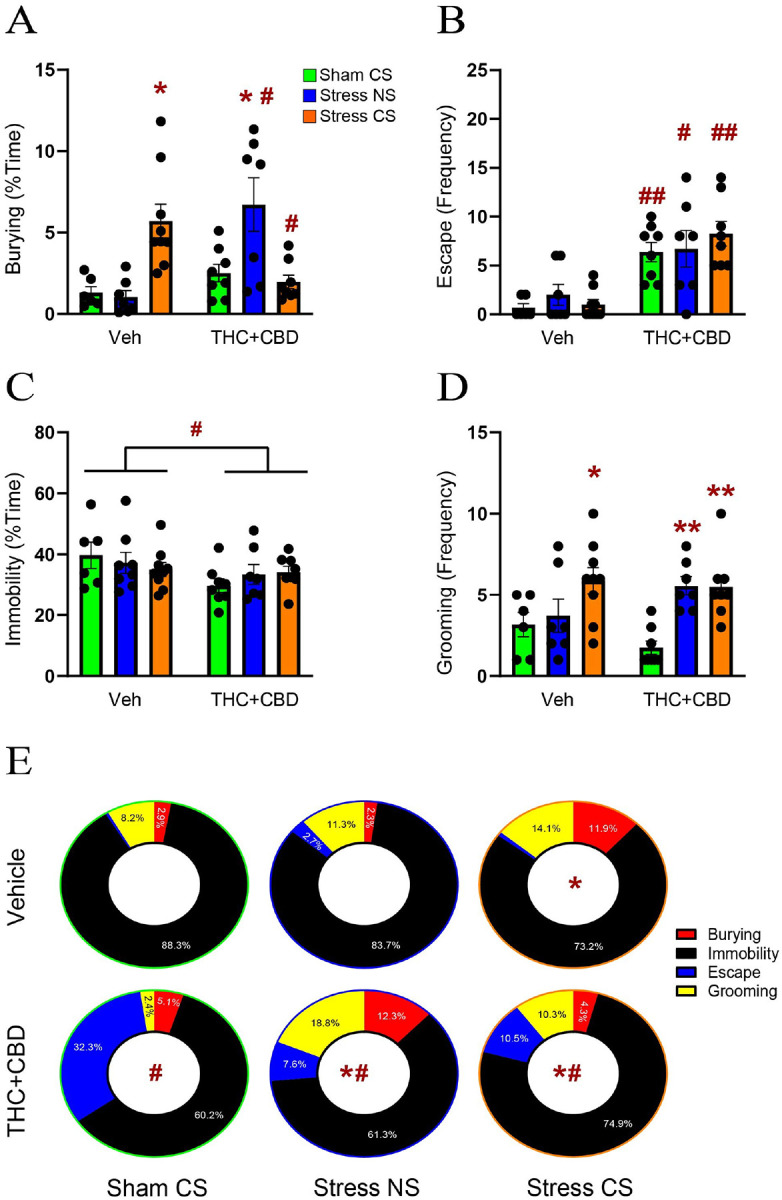
THC+CBD generalized stress responses in males. **A)** Stress-CS increased the time spent burying in vehicle-trained rats, while the Stress-NS led to an increase in the time spent burying in THC+CBD-trained rats (interaction: F_2,39_=14.77, p<0.0001). **B)** THC+CBD use generalized the escape behavior across all stress conditions (drug: F_1,39_=41.35, p<0.0001). **C)** THC+CBD reduced immobility in all three conditions (drug: F_1,39_=4.842, p=0.0338). **D)** Stress-CS enhanced grooming frequency in vehicle-trained rats, whereas both Stress-NS and Stress-CS increased grooming in THC+CBD-trained rats (stress: F_2,39_=9.835, p=0.0003). Data are shown as mean ± SEM. Each dot in bar represents one rat. **E)** Donut charts showing the distribution of the different coping strategies within each treatment. Stress-CS changed coping strategies in vehicle- (X^2^_(3)_ =34.06, p<0.0001 versus Sham-CS veh) and THC+CBD-trained rats (X^2^_(3)_ =34.06, p<0.0001 versus Sham-CS THC+CBD). However, THC+CBD promoted avoidant coping strategies during stress-CS exposure (X^2^_(3)_ =106.5, p<0.0001 versus Stress-CS veh). THC+CBD altered coping distribution in non-stressed rats (X^2^_(3)_ =974.4, p<0.0001 versus Sham-CS veh). THC+CBD modified stress responses during exposure to the neutral stimulus (X^2^_(3)_ =70.45, p<0.0001 versus stress-NS veh; X^2^_(3)_ =113.2, p<0.0001 versus Sham-CS THC+CBD). * p< 0.05, **p<0.01 compared to Sham-CS within each drug group using a Tukey or Chi-square post hoc; # p< 0.05, ## p<0.01 comparing THC+CBD to vehicle within each stress/sham group.

Following the withdrawal period, we used DBT to evaluate the effect of THC + CBD on active (burying) and avoidant (escape, immobility and grooming) coping strategies. To this end, stressed rats were exposed to either the stress-CS odor previously associated with the stressful event or a neutral odor stimulus (NS) for 15 min. Non-stressed rats were exposed to the odor previously associated with the sham experience. Regarding active coping, in vehicle-trained male rats, the stress-CS caused an increase in burying compared to the sham group. Conversely, in THC + CBD-trained rats, the stress-NS group exhibited greater burying than the sham group. Furthermore, compared to vehicle rats, THC + CBD self-administration elevated burying in the stress-NS group while decreasing it in the stress-CS group (Fig. 2A). Regarding avoidant coping strategies, we found that THC + CBD self-administration induced a strong escape behavior under all stress conditions compared to vehicle rats (Fig. 2B). Conversely, the percent time spent immobile was reduced after THC + CBD (Fig. 2C). Moreover, stress-CS significantly increased grooming in vehicle-trained rats, while both stressed groups (CS and NS) exhibited a higher grooming frequency than the sham group in THC + CBD-trained rats (Fig. 2D). When comparing the distribution of coping strategies between groups, we found no difference among the stress-NS and sham-CS groups in vehicle-trained rats (Fig. 2E), while the stress-CS promoted an active coping strategy by increasing burying behavior. In summary, Fig. 2 shows that THC + CBD withdrawal promoted avoidant coping in the sham group, while a neutral odor stimulus facilitated both active and avoidant coping in stressed rats. Also, exposure to the stress-CS after THC + CBD further changed stress behaviors compared to the sham and stress-CS veh groups. Combined, these data indicate that THC + CBD use caused male rats exposed to an earlier stressor to generalize stress responses to neutral stimuli and worsen stress responses to conditioned stimuli.

Surprisingly, in females, neither the stress nor the THC + CBD significantly affect coping strategies in the DBT (Fig. S2).

### Withdrawal from cannabinoids reduced the synaptic proximity and promoted astrocytic insulation of synapses after the stress-CS exposure in male rats

**Figure 3:**
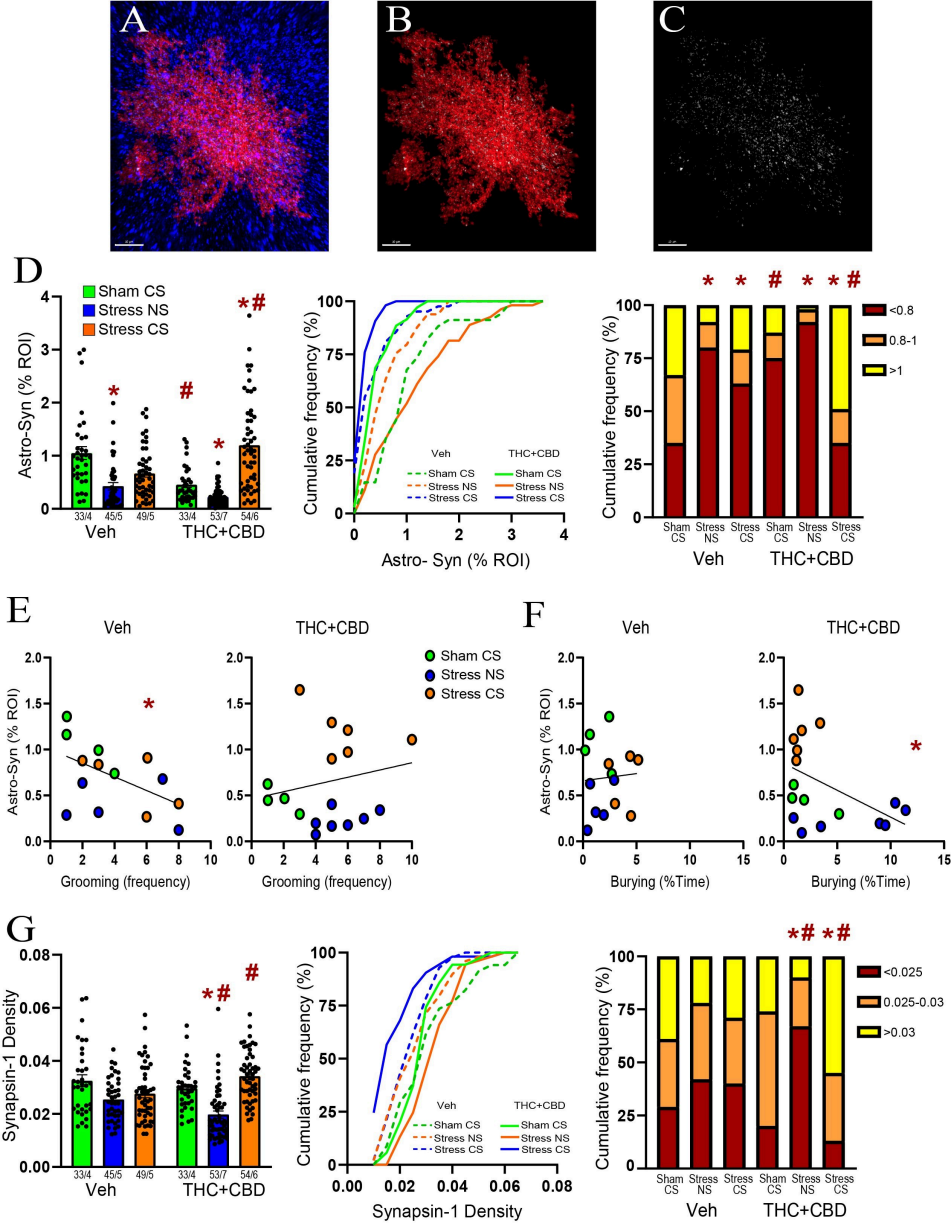
THC+CBD use reduced the astroglial coverage of synapses in NAcore but potentiated the synaptic insulation by astrocytes after the stress-CS exposure in males. **A)** Representative confocal image of an astrocyte (red) surrounded by the presynaptic marker Synapsin-I (blue). **B)** Digital rendering of the astrocyte in A. **C)** Puncta showing co-labeling between astrocyte and Synapsin-I. **D)** Left panel: Withdrawal from THC+CBD constitutively reduced astrocyte-Synapsin-I proximity, while re-exposure to the stress-CS strongly increased the colocalization between astrocytes and Synapsin-I (Kruskal-Wallis=95.97, p<0.0001). Middle panel: Cumulative frequency distribution of the astrocyte-Synapsin-I colocalization. Right panel: Stress-CS exposure did not fully restore the synaptic insulation by astroglia in vehicle-trained rats (X^2^_(2)_ =16, p=0.0003 versus Sham-CS). See table S1 for complete analysis. **E)** The negative correlation between the astrocyte-Synapsin-I colocalization and grooming frequency in vehicle-trained rats (Spearman r=−0.476, p=0.0435) disappeared following THC+CBD use. **F)** Negative correlation between the astrocyte-Synapsin-I colocalization and time spent burying in THC+CBD-trained rats (Pearson r=−0.444, p=0.0369), not in vehicle-trained rats. **G)** Left panel: Acute stress and THC+CBD withdrawal reduced, while re-exposure to the stress-CS elevated Synapsin-I density (Kruskal-Wallis=62.39, p<0.0001). Middle panel: Cumulative frequency distribution of the Synapsin-I density. Right panel: Astrocytes surrounded by higher Synapsin-I density represented a larger population in the Stress-CS THC+CBD (X^2^_(2)_ =17.5, p=0.0002 versus Sham-CS THC+CBD). Data are shown as mean ± SEM. Each dot in bar represents an astrocyte. * p< 0.05 compared to Sham-CS within each drug group using a Dunn or Chi-square post hoc; # p< 0.05 comparing THC+CBD to vehicle within each stress/sham group.

To determine how stress and cannabinoids influence the plasticity of NAcore astrocytes, astroglia were labeled with a membrane-bound fluorescent reporter. Rats were perfused immediately after DBT, and NAcore slices labeled with the presynaptic marker Synapsin-I (Fig. 3A). To quantify proximity between astrocytes and synapses, each astrocyte was digitally rendered (Fig. 3B), and the co-labeling of the astrocyte surface with Synapsin-I was quantified (Fig. 3C). Consistent with our previous report[21], we found that acute stress (stress-NS) induced a constitutive reduction in astrocyte-Synapsin-I proximity, while exposure to the stress-CS resulted in a re-association between astrocytes and Synapsin-I in vehicle-trained male rats. Withdrawal from THC + CBD decreased astrocyte-Synapsin-I proximity in the sham group compared to vehicle rats. In stressed THC + CBD-trained rats, exposure to the NS further decreased the proximity between astrocytes and Synapsin-I relative to sham, while re-exposure to the stress-CS enhanced synaptic insulation by astroglia. Additionally, when compared to vehicle rats, the use of THC + CBD potentiated astrocytic insulation of synapses induced by the stress-CS (Fig. 3D, left). Because the cumulative frequency plots (Fig. 3D, middle) indicated different subpopulations of astrocytes, we divided the data into three subpopulations based on the values generating three equal populations in the sham-CS vehicle group. The multiple Chi^2^ analysis revealed that exposure to the stress-CS did not fully restore the astrocytic insulation of synapses in vehicle-trained rats (Fig. 3D, right). Indeed, the astrocytes having less co-registration with Synapsin-I constituted a larger population in the stress-CS group than in the sham-CS group.

Astrocyte-Synapsin-I proximity negatively correlated with grooming in vehicle-trained males (Fig. 3E, left). This correlation disappeared after THC + CBD use (Fig. 3E, right). Furthermore, while no correlation existed between astrocyte-Synapsin-I proximity and burying in vehicle-trained rats (Fig. 3F, left), a negative correlation appeared after THC + CBD use (Fig. 3F, right). This illustrates that using cannabinoids alters the function of synaptic insulation in managing coping strategies.

In females, exposure to the stress-CS increased the astrocyte-Synapsin-I proximity in both vehicle- and THC + CBD-trained rats (Fig. S3A, left). Compared to vehicle rats, astrocytes near Synapsin-I were more abundant in the stress-NS group, while stress-CS exposure nearly eliminated astrocytes at mid-distance in THC + CBD females (Fig. S3A right). Moreover, in THC + CBD-trained females, astrocyte-Synapsin-I proximity correlated positively with burying behavior and negatively with grooming (Fig. S3B-C).

### Combination of acute stress and cannabinoid use reduced Synapsin-I density in NAcore

Chronic stress is known to diminish the levels of synaptic markers, such as Synapsin-I[22]. We previously found that neither acute stress nor stress-CS exposure altered Synapsin-I density in the NAcore of male rats[21]. Nevertheless, the impact of acute stress followed by cannabinoid use on Synapsin-I remains unknown. Synapsin-I density was measured for each astrocyte digitally isolated. Similar to our prior report, Synapsin-I density remained unchanged in vehicle-trained males, regardless of the stress condition (Fig. 3G, left). Likewise, no difference was found between sham-CS groups. These findings suggest that the changes in astrocyte-Synapsin-I proximity observed in vehicle-trained rats and during withdrawal from THC + CBD result solely from astrocytic motility. However, the combination of acute stress and THC + CBD withdrawal lowered Synapsin-I density in stress-NS rats, while exposure to the stress-CS restored Synapsin-I density. The multiple Chi^2^ analysis indicated that astrocytes with high Synapsin-I density formed a larger population in the stress-CS group compared to the sham-CS group (Fig. 3G, right). Furthermore, THC + CBD increased Synapsin-I density in the stress-CS group compared to the vehicle rats. This indicates that the potentiation of synaptic insulation induced by the drug following the stress-CS exposure observed in Fig. 3D could result largely from increased neuronal synapses associated with astroglia.

Unlike males, the interaction between acute stress and cannabinoid use increased the Synapsin-I density in female NAcore (Fig. S3D, left). In stressed vehicle females, astrocytes with low Synapsin-I density increased compared to the sham-CS group. These effects are reversed after THC + CBD use (Fig. S3D, right).

### Stress-CS induced astrocyte atrophy after cannabinoid use in male rats

**Figure 4:**
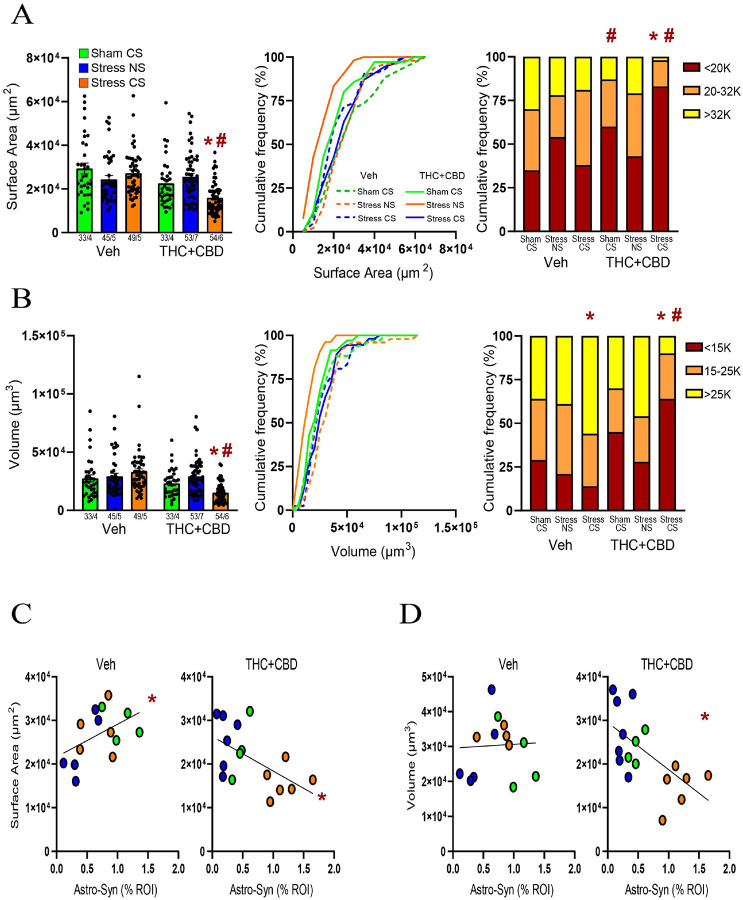
THC+CBD use induced astrocyte atrophy after the stress-CS exposure in males. **A)** Left panel: Stress-CS decreased the surface area in THC+CBD-trained rats (Kruskal-Wallis=47.67, p<0.0001). Middle panel: Cumulative frequency distribution of the surface area. Right panel: THC+CBD use increased the subpopulation of astrocytes with small surface area in Sham rats (X^2^_(2)_ =14.33, p=0.0008 versus Sham-CS Veh), which was further augmented by stress-CS exposure (X^2^_(2)_ =15.19, p=0.0005 versus Sham-CS THC+CBD). **B)** Stress-CS decreased the volume in THC+CBD-trained rats (Kruskal-Wallis=51.84, p<0.0001). Middle panel: Cumulative frequency distribution of the volume. Right panel: Stress-CS increased the subpopulation of astrocytes with large volume in vehicle-trained rats (X^2^_(2)_ =13.33, p=0.001 versus Sham-CS Veh), and the effect of stress-CS was reversed following THC+CBD use (X^2^_(2)_ =64.4, p<0.0001 versus Stress-CS Veh). **C)** THC+CBD changed a positive correlation between astrocyte-Synapsin-I colocalization and surface area (left; Pearson r=0.486, p=0.039) into a negative one (right; Pearson r=−0.512, p=0.0177). **D)** Astrocyte-Synapsin-I colocalization negatively correlated with volume following THC+CBD use (Pearson r=−0.612, p=0.0039). Data are shown as mean ± SEM. Each dot in bar represents an analyzed astrocyte. * p< 0.05 compared to Sham-CS within each drug group using a Dunn or Chi-square post hoc; # p< 0.05 comparing THC+CBD to vehicle within each stress/sham group.

Chronic stress causes astrocyte shrinkage in various brain areas[23]. Previously, we found that neither stress nor stress-CS influence astrocyte structure in the NAcore of males[21]. We replicated these negative data in vehicle males and demonstrated that after THC + CBD use the stress-CS reduced both the surface area and the volume of astrocytes (Fig. 4A–B left). Moreover, in Sham rats, THC + CBD use increased the subpopulation of astrocytes with smaller surface areas compared to Sham vehicle rats (Fig. 4A right). Additionally, in vehicle-trained rats, the stress-CS group exhibited a higher proportion of astrocytes with larger volumes than the sham group (Fig. 4B right).

Cannabinoid use in males transformed a positive correlation between surface area and astrocyte-Synapsin-I proximity in vehicle-trained rats into a negative correlation (Fig. 4C). Additionally, in THC + CBD-trained rats, the volume showed a negative correlation with astrocyte-Synapsin-I proximity, unlike in vehicle rats (Fig. 4D). In summary, these data suggest that exposure to a stress-CS interacts with cannabinoid withdrawal to induce an increase in both astroglial coverage of synapses and astrocyte atrophy.

In females, the acute stress induced a constitutive decrease in astrocyte surface area and volume in both vehicle- and THC + CBD-trained rats. Re-exposure to stress-CS restored astrocyte morphology in vehicle females, but THC + CBD prevented this effect (Fig. S4A-B). Additionally, when comparing astrocyte morphology between sexes, THC + CBD withdrawal alone increased both the surface area and volume of astrocytes in females compared to males. (Fig. S4C-D).

### MMP-2,9 activity is increased during withdrawal from cannabinoid use and is potentiated following the stress-CS exposure in male rats

**Figure 5:**
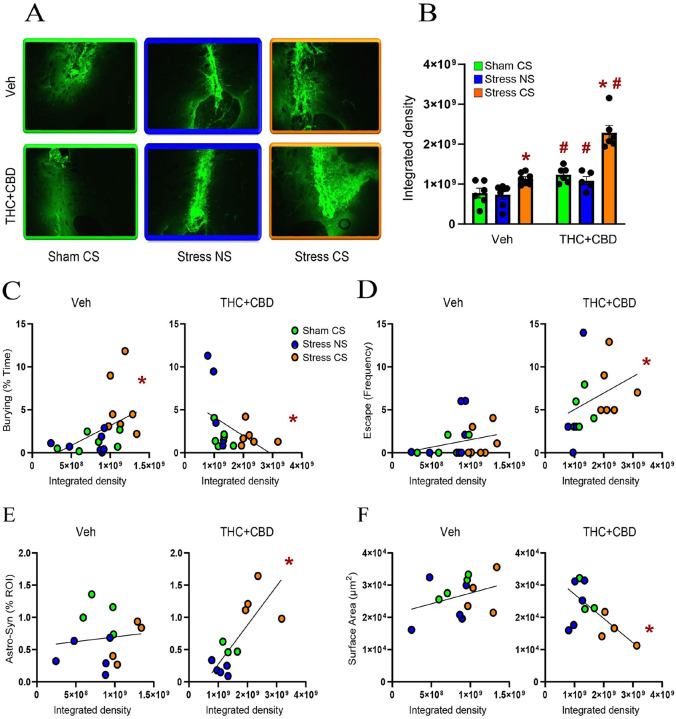
THC+CBD use potentiated the MMP-2,9 activity induced by the stress-CS in males. **A)** Representative micrograph of in vivo zymography assay from each treatment group. **B)** THC+CBD withdrawal increased MMP-2,9 activity across all stress conditions (drug: F_1,31_=54.84, p<0.0001). Stress-CS increased the MMP-2,9 activity in NAcore of both vehicle- and THC+CBD-trained rats (stress: F_2,31_=33.03, p<0.0001), but THC+CBD use exacerbated the MMP-2,9 activity (interaction: F_2,31_=7.686, p=0.0019). **C)** THC+CBD shifted a positive correlation between burying and MMP-2,9 activity (left; Pearson r=0.485, p=0.03) into a negative correlation (right; Pearson r=−0.456, p=0.03). **D)** MMP-2,9 activity positively correlated with escape behavior in THC+CBD-trained rats (Spearman r=0.54, p=0.027). **E)** MMP-2,9 activity positively correlated with astrocyte-Synapsin-I colocalization (Pearson r=0.75, p=0.0049), and **F)** negatively correlated with astroglia surface area following THC+CBD use (Pearson r=−0.547, p=0.033). Data are shown as mean ± SEM. Each dot in bar represents an astrocyte. * p< 0.05 compared to Sham-CS within each drug group using a Dunn post hoc; # p< 0.05 comparing THC+CBD to vehicle within each stress/sham group.

MMP- 2 and – 9 are key components of the ECM signaling and multipartite plasticity [16, 24]. Both addictive drugs and stress exposure alter the activity of MMP-2 and MMP-9[17]. Using in vivo zymography we quantified the gelatinolytic activity of MMP-2,9 during the DBT[19]. Withdrawal from THC + CBD increased NAcore MMP-2,9 activity regardless of the stress condition in males (Fig. 5B). Moreover, stress-CS exposure increased the MMP-2,9 activity in both vehicle and THC + CBD males. However, THC + CBD use potentiated the MMP-2,9 activity compared to vehicle rats.

Cannabinoid use reversed a positive correlation between MMP-2,9 activity and burying behavior in vehicle-trained rats into a negative correlation (Fig. 5C). Moreover, MMP-2,9 activity was positively correlated with the escape behavior in THC + CBD-trained, not vehicle rats (Fig. 5D). These data indicate that cannabinoid use modifies how ECM signaling regulates coping strategies, specifically by inducing a shift from active to avoidant coping. Additionally, after cannabinoid use, MMP-2,9 activity positively correlated with astrocyte-Synapsin I proximity, while negatively correlating with astrocyte surface area (Fig. 5E–F), suggesting that ECM signaling regulates the astroglial plasticity.

Neither stress nor the THC + CBD alter the MMP-2,9 activity in females (Fig. S5A). However, similar to males, MMP-2,9 activity showed a positive correlation with astrocyte-Synapsin-I proximity and a negative correlation with astrocyte volume after cannabinoid use (Fig. S5B-C).

### Both MMP-2 and MMP-9 regulated the astroglial coverage of synapses, while MMP-2 influenced the astrocyte morphology

**Figure 6:**
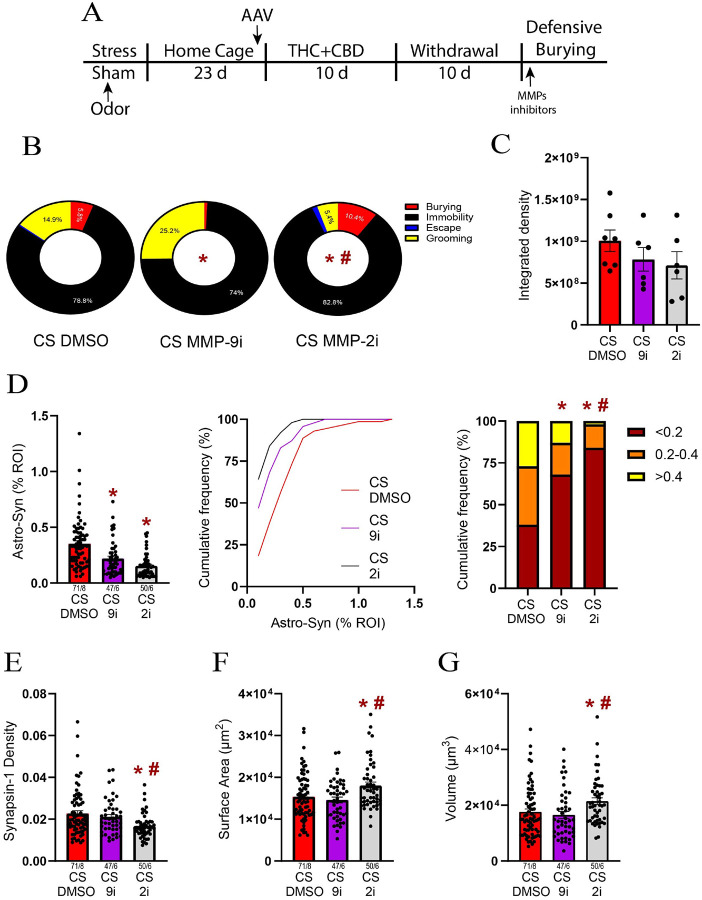
MMP-2 regulated the multipartite synaptic plasticity following THC+CBD use. **A)** Experimental timeline outlining THC+CBD self-administration, withdrawal and the defensive burying task. **B)** Both MMP-2i (X^2^_(2)_ =10.6, p=0.005 versus DMSO) and MMP-9i (X^2^_(2)_ =10.32, p=0.0058 versus DMSO) changed coping responses during exposure to the stress-CS. Moreover, MMP-2i also changed the coping distribution compared to MMP-9i (X^2^_(2)_ =96.79, p<0.0001). **C)** MMP-2i and MMP-9i did not alter the MMP-2,9 activity. **D)** Left panel: Both MMP-2i and MMP-9i reduced the astrocyte-Synapsin-I colocalization. Middle panel: Cumulative frequency distribution of the astrocyte-Synapsin-I colocalization. Right panel: MMP-9i increased the subpopulation of astrocytes located farther from Synapsin-I (X^2^_(2)_ =18.13, p=0.0001 versus DMSO), while MMP-2i nearly eliminated astrocytes located near Synapsin-I (X^2^_(2)_ =10.51, p=0.0052 versus MMP9i). **E)** MMP-2i reduced the Synapsin-I density (Kruskal-Wallis=12.32, p=0.0021). **F)** MMP-2i increased astroglia surface area (Kruskal-Wallis=9.37, p=0.0092). **G)** MMP-2i increased astroglia volume (Kruskal-Wallis=11.28, p=0.0035). Data are shown as mean ± SEM. Each dot in bar represents an astrocyte. * p< 0.05 compared to DMSO using a Dunn or Chi-square post hoc; # p< 0.05 comparing 9i to 2i.

To determine whether the changes in astroglial plasticity induced by the stress-CS in males are preceded by changes in the ECM catalytic signaling, we examined the effect of MMP-2 and MMP-9 inhibitors on stress-CS-induced astroglial plasticity. To this end, during THC + CBD withdrawal, another cohort of stressed males received bilateral microinjections of inhibitors (0.1 nmol) or their vehicle (1% DMSO) in NAcore, followed by a unilateral microinjection of the FITC-gelatin before stress-CS exposure (Fig. 6A). Compared to the DMSO microinjection, either MMP-2 or MMP-9 inhibitors altered the coping strategies. MMP-9 inhibitor notably reduced the burying behavior (Fig. 6B and S6). The coping strategies also significantly differ between the MMP-2 and MMP-9-treated groups (Fig. 6B), with the MMP-2 inhibitor significantly reducing grooming (Fig. 6B and S6). Interestingly, these alterations in stress-CS-induced behaviors were accompanied by only a nonsignificant reduction in gelatinolytic activity by either of the MMP inhibitors (Fig. 6C).

Both MMP-2 and − 9 inhibitors reduced the astroglial coverage of synapses (Fig. 6D left). Furthermore, the MMP-2 inhibitor nearly eliminated astrocytes found near Synapsin-I (Fig. 6D right). A likely contributor to the reduction in synaptic coverage by astroglia was markedly reduced Synapsin-I density after treatment with the MMP-2, but not MMP-9 inhibitor (Fig. 6E). Finally, the MMP-2 inhibitor reversed astrocyte atrophy by increasing both surface area and volume (Fig. 6F–G).

## Discussion

We recently demonstrated that cannabis use, after a single traumatic event, promotes two PTSD-like symptoms: generalization of stress responses to neutral stimuli and avoidant coping strategies following conditioned stress exposure[11]. To explore the neuroadaptations behind these changes, we used in vivo zymography and confocal microscopy to assess how stress and cannabinoid use affect multipartite synaptic plasticity in the NAcore, a brain region linked to stress and addiction cues responding[2]. We made four primary discoveries: 1) cannabinoid withdrawal, without any traumatic event, reduced the astroglial coverage of synapses and increased MMP-2,9 signaling in the ECM, 2) a prior traumatic event reduced Synapsin-I density during cannabinoid withdrawal, 3) exposure to a stress-CS after cannabinoid use led to avoidant coping strategies by activating MMP-2,9 in the NAcore, resulting in the re-association of astrocytes with synapses and astrocyte atrophy, and 4) these multipartite adaptations by stress and cannabinoids occurred only in males.

### THC + CBD disrupts coping strategies in males

In line with our previous report, we found that THC + CBD use generalized stress responses to a neutral stimulus in stressed rats, by inducing both active (burying) and avoidant (escape and grooming) coping strategies. Importantly, a neutral stimulus did not trigger stress responses in vehicle-trained rats, suggesting that the effects seen in THC + CBD-trained rats result from the interaction between acute stress and cannabinoid withdrawal. Additionally, we found that cannabinoid use induced notable escape behavior in non-stressed rats. Both preclinical and clinical studies support a contention that extensive and prolonged cannabis use is associated with more severe withdrawal symptoms[25–27] and our data indicate that cannabinoid use, regardless of previous traumatic experiences, generalizes stress responses to unconditioned stimuli. Since stress generalization is a hallmark of PTSD[1], these preclinical data support longitudinal clinical studies[5, 6] indicating that cannabis use worsens PTSD symptoms. Furthermore, we previously demonstrated that a stress-CS is sufficient to reinstate cocaine, heroin, and alcohol seeking, reflecting an active coping strategy[12, 14]. Accordingly, we found that the stress-CS induced burying behavior in vehicle-trained rats. However, after cannabinoid use, re-exposure to the stress-CS reduced burying while promoting escape and grooming behaviors. Since avoidance behavior is a key criterion for defining PTSD, and avoidance coping strategies are associated with increased activation of the stress axis[28], our findings suggest that cannabis use heightened the salience of the stress-CS and thereby worsened PTSD symptoms.

### THC + CBD use alters how astroglial coverage regulates coping strategies in males

The synaptic insulation provided by astrocytes plays a crucial role in synaptic plasticity by supplying metabolic support, gliotransmitters, and maintaining ion homeostasis[16]. Withdrawal from cocaine, heroin, and methamphetamine self-administration reduced the proximity between astrocytes and synapses in NAcore[29–32]. Similarly, we demonstrated that withdrawal from THC + CBD reduced the astroglial coverage of synapses in NAcore, suggesting that this effect is shared by many classes of addictive drugs. Akin to withdrawal from addictive drugs, an acute restraint stress also induced enduring decreases in astroglial coverage in NAcore[21]. Consistently, we found that acute stress reduced astrocyte-Synapsin-I proximity in both vehicle- and THC + CBD-trained rats. Finally, we demonstrated that re-exposure to the stress-CS increased the astroglial coverage of synapses in both vehicle- and THC + CBD-trained rats. However, while the stress-CS only partially restored the astrocytic insulation of synapses in vehicle-trained rats, cannabinoid exposure potentiated the re-association of astrocytes to synapses induced by the stress-CS. In examining astroglial insulation related to coping strategies, we found a negative correlation between astrocytes-Synapsin-I proximity and grooming behavior in vehicle-trained rats. This implies that astroglial coverage may partly countermand the expression of avoidant coping behaviors, although this correlation vanished following THC + CBD usage. Conversely, in THC + CBD-trained males, the proximity of astrocytes to Synapsin-I was negatively correlated with burying behavior. Thus, THC + CBD inhibits active coping strategies and potentially exacerbates the salience of the stress-CS by promoting synaptic insulation by astrocytes. Overall, this finding suggests that stress-CS exposure during cannabinoid withdrawal alters how astroglial coverage in the NAcore influences coping strategies.

### THC + CBD use following an acute stress reduces the Synapsin-I density in NAcore

Severe stressors are known to decrease the levels of synaptic markers, including Synapsin-I and PSD95, in several brain regions. For example, chronic unpredictable mild stress (CUMS) decreases the expression of both Synapsin-I and PSD95 in the prefrontal cortex and hippocampus[22, 33] and a single prolonged stress (SPS) reduces Synapsin-I and PSD95 in the hippocampus[34]. In NAcore, the combination of SPS and foot shock reduces PSD95[35]. In contrast, we found that neither 2 hours of acute restraint stress, stress-CS exposure, nor withdrawal from THC + CBD without stress pre-exposure affected the Synapsin-I density in the NAcore[7]. Thus, the reduction seen in astroglial coverage produced by stress pre-exposure (stress-NS) or THC + CBD without stress pre-exposure are largely due to astrocytic motility not an overall reduction in synapses. In contrast, we found that both Synapsin-I and astroglial synaptic proximity were reduced in the group receiving acute restraint stress and cannabinoid use (Stress-NS). Thus, the retraction of astrocytes induced by cannabinoid withdrawal, along with the loss of synapses resulting from the interplay between stress and cannabinoid use, may contribute to how THC + CBD generalizes stress responses to a neutral stimulus.

### THC + CBD use induces astrocyte atrophy during exposure to the stress-CS

It is well-documented that stress alters astrocyte morphology[23]. For example, chronic restraint stress reduced the process length, branching, and volume of astrocytes in the prefrontal cortex[36], and astrocyte volume in the amygdala[37]. CUMS also induced astrocyte atrophy in the prefrontal cortex and hippocampus[38, 39]. Moreover, the SPS protocol led to astrocyte atrophy in the hippocampus[40], where astrocyte atrophy is linked with anxiety and depressive-like behavior. For instance, the astrocyte morphology negatively correlates with anxiety, while treatments restoring the astrocyte morphology alleviate symptoms of anxiety and depression in animal models[39–41]. In NAcore, neither acute restraint stress nor the stress-CS affect astroglial morphology[21].

In contrast with stress, the effects of THC on astrocyte morphology are less understood. In organotypic hippocampal slices, prolonged incubation with THC reduces astrocytic processes[42]. Astrocytic processes are also reduced in the amygdala of rats treated with high doses of THC during adolescence[43]. However, the effects of voluntary cannabinoid consumption on astrocyte structure were unknown. Here, we demonstrated that THC + CBD self-administration, with or without a prior stressful experience, did not affect measures of astrocyte morphology, including surface area and volume. However, re-exposure to the stress-CS triggered astrocyte atrophy in THC + CBD, not vehicle-trained rats. Surprisingly, we observed a negative correlation between astrocyte surface area and volume and synapse coverage, indicating that cannabinoid use and stress-CS triggered both astrocyte atrophy and increased proximity of astroglia to synapses.

### THC + CBD use changes the function of MMP-2,9 in regulating coping strategies in males

The gelatinases MMP-2 and MMP-9 play a necessary role in activity-dependent multipartite synaptic plasticity[16]. For example, MMP-9 controls dendritic spine enlargement induced by long-term potentiation[44]. Thus, over the last 20 years, research has focused on the role of MMP-2,9 in various psychiatric disorders, including drug and stress-related disorders[17]. Our laboratory demonstrated that drug seeking induced by Pavlovian cues requires transient activation of MMP-9 in the NAcore, which leads to both spine head expansion and the insertion of AMPA receptors on D1-medium spiny neurons[20, 45, 46]. In parallel, withdrawal from cocaine or heroin self-administration constitutively increases the MMP-2 activity around D2-MSNs [20, 47]. Concerning stress-related disorders, both acute and chronic restraint stress increase MMP-9 activity in the hippocampus[48, 49]. We also found that acute restraint stress increases MMP-9 in NAcore[19]. However, the effect of stress-CS on the activity of MMP-2,9 was unknown. Given that both stress-CS and drug cues promote spine head expansion[11, 45], we hypothesized that stress-CS would increase MMP-2,9 activity. As anticipated, MMP-2,9 activity was increased after exposure to stress-CS in vehicle-trained rats. We also demonstrated that THC + CBD withdrawal constitutively increased MMP-2,9 activity. The combination of stress pre-exposure and cannabinoid withdrawal (stress-NS) did not alter the basal activity of MMP-2,9. However, re-exposure to the stress-CS during cannabinoid withdrawal increased the MMP-2,9 activity. Cannabinoid use not only elevated MMP-2,9 activity but also contributed to a shift from active to avoidant coping behaviors. Thus, while MMP-2,9 activity positively correlated with burying in vehicle-trained rats, cannabinoid use reversed the positive correlation to a negative correlation with burying and revealed a positive correlation with escape behavior.

### MMP-2 is a key regulator of the multipartite synaptic changes induced by the stress-CS following cannabinoid use.

Considering that stress-CS re-exposure after cannabinoid use strongly activated MMP-2,9 and simultaneously increased astroglial coverage and astrocyte atrophy, we hypothesized that these events are interconnected. Supporting our hypothesis, MMP-2,9 activity positively correlated with astroglial coverage and negatively with astrocyte surface area. To establish a causal relationship between ECM catalytic signaling and astroglial plasticity, we administered inhibitors of both gelatinases before the DBT. We found that inhibiting either MMP-2 and MMP-9 reduced astroglial coverage of synapses in NAcore, while only the MMP-2 inhibitor reduced Synapsin-I density and prevented astrocyte atrophy. Additionally, inhibiting MMP-2 decreased grooming while increasing burying, indicating that MMP-2 activation by stress-CS contributes to the switch from active to avoidant behaviors in THC + CBD withdrawn rats. In summary, these data constitute the first in vivo evidence linking the catalytic activities of MMP-2 and MMP-9 to astroglial plasticity and stress-associated behaviors.

### Sexual dimorphism

Women are twice as likely to develop PTSD and exhibit greater vulnerability for anxiety-related disorders [51–53]. However, historically, preclinical studies on stress-related disorders mostly used males and focused primarily on freezing behavior[50]. Including females and assessing other stress responses introduced new insights into coping strategies[51, 52]. Indeed, research demonstrates that male rats freeze more than females, while females display higher escape-like behaviors[50, 53–55]. Accordingly, we found that females escape more than males across all stress conditions. Surprisingly, neither stress nor THC + CBD had a significant impact on coping strategies in females. This may be due to high variability we observed in stress responses between females. Although the estrous cycle is not involved in escape-like behavior [42], it would be interesting to evaluate its influence on other coping responses. Despite not affecting behaviors, stress and cannabinoid use changed multipartite plasticity in females. Unlike males, the number of synapses (Synapsin-I puncta) is increased in stressed rats following THC + CBD use. Regarding astroglial plasticity, cannabinoid withdrawal without stress pre-exposure increased both the surface area and volume of NAcore astrocytes in females compared to males. This result differs from cocaine, where prolonged withdrawal elicits NAcore astrocyte atrophy in males without affecting female astrocytes[29]. Furthermore, while acute restraint stress did not affect astrocyte morphology in males, it induced astrocyte atrophy in females. The opposite effect is observed after chronic stress, where CUMS reduces astrocyte morphology in males without affecting females[56]. Our findings contribute to research on sexual dimorphism in astroglial plasticity. More studies are needed to understand how stress and cannabis differently affect female and male multipartite plasticity.

### Clinically relevant targets

This study identified neurobiological mechanisms behind PTSD-like symptoms after cannabinoid use in male rats, highlighting potential therapeutic targets such as MMPs and astrocytes for comorbid CUD/PTSD. Recent studies demonstrate elevated plasma levels of MMP-2 and MMP-9 in PTSD patients[57, 58]. Moreover, the nonselective MMP inhibitor doxycycline disrupts the consolidation of cued fear memory in humans[59]. Additionally, a single dose of minocycline, a more specific MMP-9 inhibitor, administered before a contextual fear memory paradigm, reduced the consolidation of fear memory[60]. These clinical data suggest that MMP inhibition represents a potentially effective strategy to treat the comorbidity between PTSD and CUD. Another interesting strategy would be to target astroglial plasticity. For example, the antidepressant fluoxetine alleviates depressive-like symptoms, partly by reversing astrocyte atrophy caused by CUMS[39]. Moreover, in humans, antidepressant treatment reverses astroglial atrophy in the hippocampus[61]. Thus, exploring the impact of antidepressant treatment on astrocyte atrophy resulting from stress-CS after cannabis use would be intriguing.

## Supplementary Material

Supplementary Files

This is a list of supplementary files associated with this preprint. Click to download.

- FigS1.jpg
- FigS2.jpg
- FigS3V1.jpg
- FigS4V1.jpg
- FigS5V1.jpg
- FigS6.jpg
- TableS12025.docx
